# The suitability of chili pepper (*Capsicum annuum* L.) for alleviating human micronutrient dietary deficiencies: A review

**DOI:** 10.1002/fsn3.790

**Published:** 2018-10-08

**Authors:** Tomi L. Olatunji, Anthony J. Afolayan

**Affiliations:** ^1^ Department of Botany Medicinal Plants and Economic Development (MPED) Research Centre University of Fort Hare Alice South Africa

**Keywords:** *Capsicum annuum*, chili pepper, human dietary deficiency, micronutrients, vegetables

## Abstract

Human micronutrient dietary deficiency remains an enormous global problem and probably accounts for the cause of many chronic health conditions and diseases. Above two (2) billion individuals on the planet today have been estimated to be deficient in major minerals and vitamins, predominantly zinc, iodine, vitamin A, and iron primarily due to inadequate dietary intake. The eradication of deficiencies in micronutrient on a sustainable basis will be conceivable only when diets of vulnerable populace provide all required nutrients in adequate amounts. Among the numerous approaches toward eradicating human dietary deficiency, feeding on a wide range of foods, especially vegetables that have an array of micronutrients, is still perceived as the best sustainable solution. The universal consumption of chili peppers (*Capsicum annuum*), known for their high nutritional content (which includes a good range of vitamins, minerals, phytochemicals, and dietary fiber), may play a role in decreasing human micronutrient deficiencies. Significant portions of recommended daily nutrients could be supplied by the incorporation of nutrient‐rich chili pepper into human diets which could help in combating nutrient deficiencies. This present review, therefore, gives an overview of the universal occurrence of micronutrient deficiency. It also discusses approaches that have been used to tackle the situation while stressing the potentials of chili pepper as a promising vegetable which could be utilized in alleviating human micronutrient dietary deficiencies. For all available information provided, research databases (Science direct, Academic journals, PubMed, and Google Scholar) were searched independently using keyword search strategy. Titles and abstracts were examined initially, and full papers were retrieved if studies met the inclusion criteria.

## 1 INTRODUCTION

Micronutrients, which include minerals, vitamins, antioxidants, phytochemicals, and trace elements, are indispensible for promoting good health. In contrast to macronutrients which include fat, protein, and carbohydrate, micronutrients are needed in smaller amounts and this is why they are known as “micro” nutrients (Graham et al., 2007; Regan, Keith, & Robert, 2015). Micronutrients play vital roles in normal development and growth starting from the early phase of life, and therefore, a child must consume considerable amount of the essential nutrients for the maintenance of vital processes. These indispensible micronutrients perform a range of distinctive metabolic roles as cosubstrates or cofactors in the absorption and breakdown of carbohydrates, amino acids, proteins, and lipids and to release energy and others serve as structural components of enzymes or as catalytic centers or other macromolecules (Brätter, Negretti de Brätter, Recknagel, & Brunetto, 1997; Guerrant, Lima, & Davidson, 2000; Kapil & Bhavna, 2008). Micronutrients must be obtained directly in sufficient amounts from human diets since they cannot be synthesized by humans (Kapil & Bhavna, 2008).

Micronutrient deficiencies can arise from inadequate access to foods rich in them as well as from inadequate digestion, inappropriate food processing methods leading to the destruction of micronutrients in them coupled with their mal‐absorption or postabsorptive utilization. In many developing countries, the occurrence of micronutrient deficiency has been attributed to be the major cause of poor and pitiable human health (Black et al., 2008). The most sustainable and practicable approach to alleviate these deficiencies is by integrating foods such as vegetables, livestock products, and fruits that are micronutrient‐rich into diets. Historically, vegetables are well thought to be rich sources of various indispensible micronutrients and dietary fibers, and in recent times, they have been considered as good sources for various plant chemicals that when used singly, or in combination, promote human health (Al‐Mamun et al., 2016; Flyman & Afolayan, 2006; Grubben et al., 2014; Rechkemmer, 2001). With respect to unit of land area and unit production cost, vegetables are also efficient means of numerous essential micronutrients (Ali & Tsou, 1997; Al‐Mamun et al., 2016). Among the various vegetables available throughout the world, peppers, also known as chilies, are largely considered a balanced source of most essential nutrients. Chilies have been recognized to be good sources of minerals, provitamin A, vitamins C and E, carotenoids, and phenolic compounds, metabolites with renowned antioxidant properties which influence human health positively (Materska & Perucka, 2005; Sun, Powers, & Tang, 2007). Integrating a pepper‐rich diet can therefore be helpful in the continuing quest to alleviate micronutrient deficiencies.

## 2 THE GRAVE PROBLEM OF MICRONUTRIENT DEFICIENCY

Micronutrient deficiency also called human dietary deficiency is among the most predominant and dangerous health conditions of the 21st century and it is among the foundation cause of the many chronic diseases and health conditions plaguing the earth these days (Shetty, 2011). While many individual still consider that these deficiencies only occur in underdeveloped nations, actually micronutrient deficiency is a worldwide pandemic, in each country of the planet. More than 2 billion individuals on the planet today are deficient in major minerals and vitamins, notably iodine, iron, zinc, and vitamin A. These deficiencies occur once individuals lack access to foods that are rich in micronutrients such as vegetables, fruits, fortified foods, and animal products, generally on the ground that they are excessively costly, making it impossible to purchase or are domestically inaccessible. Deficiency in micronutrients promotes morbidity and mortality from infectious illnesses such as malaria, diarrhea, measles, and pneumonia, and these conditions are one of the 10 primary causes of global health burdens (WHO, 2001). The people most susceptible to micronutrient deficiencies are expectant women, lactating mothers and their kids as a result of their comparatively greater physiological requirement for minerals and vitamins and are therefore, more exposed to the detrimental consequences of these deficiencies (Berti et al., 2011; Regan et al., 2015; Shetty, 2011). According to Howart (2000), in developing nations, above three billion individuals are deficient in iron and this is more severe in children and women due to the fact that they have more physiological requirement for iron. In resource‐constrained nations, over 50% of expectant women and over 40% of nonexpectant women as well as kindergarten kids regularly experience anemic conditions at one stage or the other in their lives. Deficiencies of iron in the course of childhood and adolescence decrease growth, mental advancement, and capacity to learn (Berti et al., 2011). In adults, deficiency of iron decreases the ability to perform physical work (Victoria et al., 2008), and it is also majorly responsible for death in pregnant women during delivery.

Universally, about three million kids at kindergarten level have visual defect as a result of deficiency in vitamin A; yearly, about 250,000 to 500,000 kindergarten age kids become blind as a result of vitamin A deficiency while nearly 65% of these children die within a short period of becoming blind (Bhutta, Salam, & Das, 2013; Howart, 2000). Subclinical occurrence of lack of vitamin A assessed ranged from 100 between 250 million as reported by Bhutta et al. (2013) and Howart (2000).

In developing countries, various clinical trials have revealed that distribution of encapsulated vitamin A can decrease death rates in preschool age kids up to 30%. In the world, iodine deficiency has been the sole cause of avertible mental retardation and brain damage (Black et al., 2008; Howart, 2000; McLean, Egli, de Benoist, & Wojdyla, 2007), and over 2 billion individuals on earth reside in areas that are iodine‐deficient. At the later stage of infancy and childhood, observable iodine deficiencies have been implicated in delayed motor development, growth delay, mental retardation, speech and hearing defects, and neuromuscular disorders (Flyman & Afolayan, 2006; Gletsu‐Miller & Wright, 2013; World Health Organization, 2000). Even mild deficiency in iodine is been stated to decrease intelligence quotients up to 10–15 points. Deficiencies in numerous other micronutrients, particularly, zinc may be equally prevalent, with similar severe deleterious effects on human health (Bhutta et al., 2013; Howart, 2000).

## 3 STRATEGIES TO TACKLE MICRONUTRIENT DEFICIENCY

Human micronutrient dietary deficiencies are highly prevalent, and therefore, action must be taken because they result to huge health, social, and economic lost. Because it is a global problem and poses various challenges, different intervention approaches have been structured in developing nations to ameliorate the present situation with respect to micronutrient deficiency of their populations as reported by Regan et al. (2015).

Time‐tested approaches globally adopted to tackle micronutrient malnutrition have up till now focused on supplementation, fortification, and dietary modification (food‐based approaches) of commonly consumed foods with micronutrients (Gibson, 2011; Shetty, 2011; Thompson & Amoroso, 2011). Considerably, efforts to tackle the three main micronutrient deficiencies of general health concern, that is, iron, iodine, and vitamin A deficiencies, have centered on supplementation. This is a specialized approach in which nutrients are directly delivered with the use of pills or syrup and is most proper for targeted populace with higher risk of deficiency or under exceptional situations, such as during pregnancy or in intense food shortage (Cristiana, Mieke, & Cornelius, 2014). In several instances, micronutrients’ provision has brought about quantifiable improvements on health, such as decreased morbidity, increased growth in kids, and decreased mortality before and after childbirth (Black, 2003; Rivera, Hotz, Gonzales‐Cossio, Neufeld, & Garcia‐Guerra, 2003). In any case, supplementation of micronutrients in tablets or other forms needs a functional healthcare framework by which dispersal and delivery take place, and also comprehension and commitment from people who partake (Haider & Bhutta, 2006).

In remote and rural regions and low‐income nations specifically, these requirements are not generally effortlessly met (Vijayaraghavan, 2002). Furthermore, when using micronutrient supplements, there are invariably dangers of over‐dose with related severe health implications as reported by Rioux and LeBlanc (2007). Moreover, formulation of micronutrient mixtures can be in fact technically tough especially when numerous trace elements need to be incorporated. Various studies have uncovered negative interactions between zinc and iron when administered at the same time (Baqui et al., 2003; Lind, Lönnerdal, & Stenlund, 2003; Penny et al., 2004). It must be stated that, under some situations, micronutrient supplementation can result in dangerous effect on the health dangerous, as reported in the case with supplementation of iron in malaria‐prone areas (Zimmermann & Hurrell, 2007). On the premise of the above‐mentioned concerns, extensive micronutrient supplementation programs become necessary, and alternative approaches, subject to prevailing local conditions, may be a better approach.

Another extensively used approach to tackle human micronutrients deficiencies is by fortifying frequently consumed foods with one or several micronutrients. This is done by the supply of iodine‐enhanced table salt, milk fortified with iron, sugar fortified with iron, and flours fortified with numerous micronutrients (copper, zinc, iron), etc. (Allen, de Benoist, Dary, & Hurrell, 2006; Lynch, 2005; Sunil, 2013). Nonetheless, this approach of micronutrient‐fortified food has its own restrictions and problems. Principally, during the process of fortification, food properties such as color, smell, and taste may be altered thus affecting the acceptability of the product for human consumption (Mannar & Gallego, 2002; Moritteo, Lee, Zimmermann, Nuessli, & Hurrell, 2005). Another major concern is the adverse interactions that result once foods are fortified with several micronutrients, thus affecting the bioavailability of micronutrients in the food fortified (Anon., 2007; Rosado, 1999). Additionally, it is only when the range of intake is not too wide that fortification of a selected food becomes safe and efficient.

Food‐based approaches to tackle human dietary deficiency encourage feeding on foods that are micronutrient‐rich or are enhanced by fortification. Increasing access to and availability as well as consuming a wide variety of foods rich in micronutrients do not just positively affect micronutrients status but also give rise to better nutrition generally. Besides its inherent nutritional value, food has economic and social importance that, for countless individuals, particularly people in developing nations, is usually facilitated by agriculture and associated activities which help in sustaining the livelihoods of rural dwellers (Rechkemmer, 2001; Stavric, 1994). The numerous health, economic, and social benefits attributed to effective food‐based approaches resulting in availability year‐round, accessibility, and feeding on nutritionally sufficient varieties and quantities of food are outstanding as the nutritional wellness and health of people are advanced, livelihoods and earnings supported, and creation and protection of national and community wealth (Cristiana et al., 2014).

Vegetables and fruits are rich in minerals and vitamins and are essential components of a balanced diet (Ali & Tsou, 1997; Al‐Mamun et al., 2016; Rai et al., 2012). They are good sources of several minerals, vitamins, dietary fiber, and phytochemicals, and they perform a critical role in averting and controlling deficiencies in micronutrient, including vitamins A, C, B (folate), and E deficiencies. Vegetables give the cheapest natural source of health‐advancing micronutrients (Al‐Mamun et al., 2016; Grubben et al., 2014). As a result of high amounts of vitamin C they contain, vegetables alleviate deficiency of iron by increasing uptake of nonhem iron in foods obtained from plants (Ali & Tsou, 1997; Rai et al., 2012). Leafy and fruit vegetables, including indigenous vegetables, have high provitamin A carotenoids content that can be changed into an active form of vitamin A by the human body. Provitamin A‐rich vegetables can make a valued contribution to intake of vitamin A and also meliorate the status of vitamin A in kids from countries where fortified foods or foods from animal source are rarely eaten or away from the reach of poor individuals (Al‐Mamun et al., 2016; Grubben et al., 2014; Rai et al., 2012).

Among the diverse vegetables available in the world, chili peppers (*Capsicum annuum*), which are rich in provitamin A, vitamins E, C, and some minerals, could give rise to meaningful improved nutrition (Materska & Perucka, 2005; Sun et al., 2007).

## 4 THE GENUS *Capsicum* AT A GLANCE

**Figure 1:**
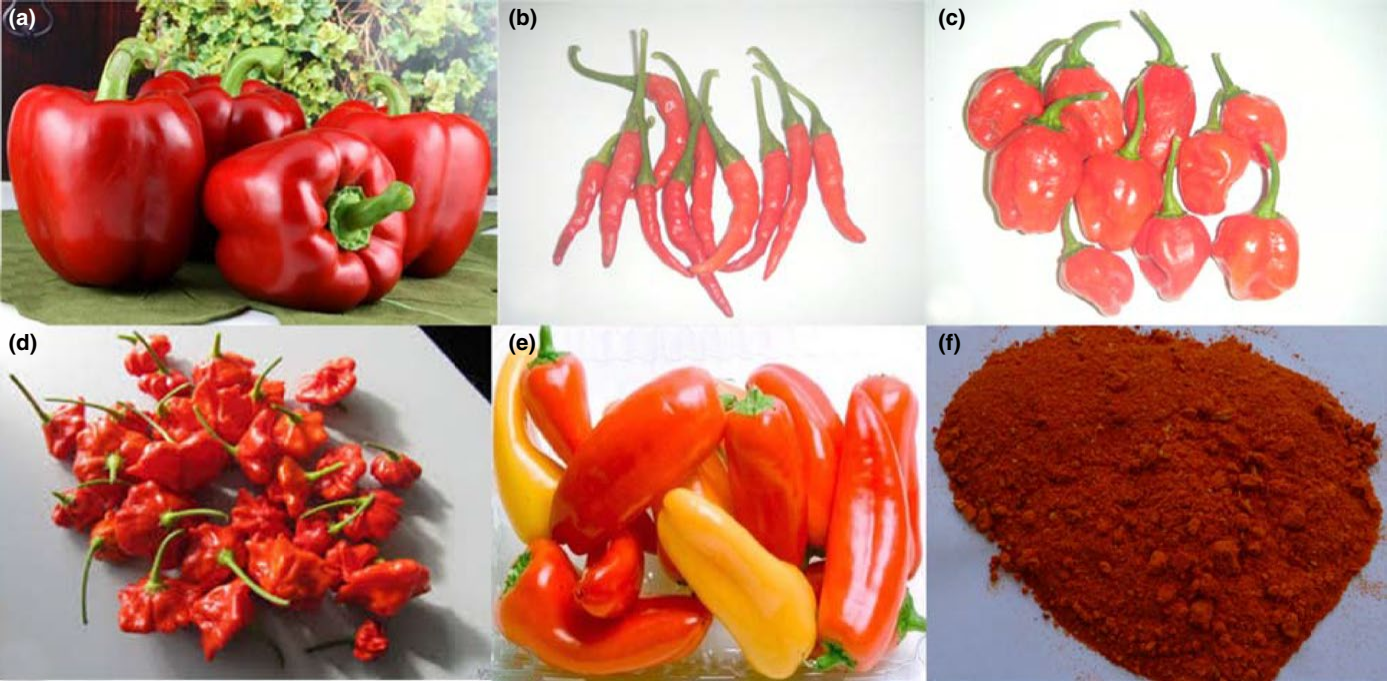
Images of the five cultivated *Capsicum* species (a) *Capsicum annuum* (b) *Capsicum frutescens* (c) *Capsicum chinense* (d) *Capsicum baccatum* (e) *Capsicum pubescens* (f) Ground pepperSource: Wikipedia

The genus *Capsicum* with several universal English names which include hot pepper, chili pepper, chili, bell pepper, chili, and sweet pepper belongs to the family *Solanaceae*. Sometimes, the plant is just called pepper. Approximately, this genus consists of five domesticated species and twenty‐two (22) wild species. Those that are domesticated are *C*. *annuum* L., *Capsicum Chinenses* Jacqs., *Capsicum frutescens* L., *Capsicum pubescens* R., and *Capsicum baccatum* L. (Table 1, Figure 1; Bosland & Votava, 2000). *Capsicum* species can be sorted into different classes based on pod or fruit features such as shape, size, pungency, flavor, and color. In spite of their enormous trait variations, the commercially cultivated cultivars of peppers globally are *C*. *annuum* species (Bosland & Votava, 2000; Smith, Villalon, & Vlla, 1987).

**Table 1:** Scientific classification of genus *Capsicum*

Kingdom	Plantae
Clade	Angiosperms
Clade	Eudicots
Clade	Asterids
Order	Solanales
Family	*Solanaceae*
Subfamily	Solanoideae
Tribe	Capsiceae
Genus	*Capsicum*
Species	*C. annuum*
	*C. frutescens*
	*C. baccatum*
	*C. pubescens*
	*C. chinenses*

Ecologically, *Capsicum* species are perennial shrubs in tropical regions (that can survive up to a decade in some cases), and they are commonly grown as annual and herbaceous plant in temperate regions and can also be cultivated as perennials in greenhouses that are climate‐regulated (Grubben & El Tahir, 2004). *Capsicum* spp. are diploids, mostly having 24 chromosomes (*n *= *x *= 12) and numerous wild species consisting of 26 chromosomes (*n *= *x *= 13). The domesticated species belong to the first group (Tong & Bosland, 2003). Like most other plants, peppers have a preference for well‐drained moisture‐holding loamy soil having an optimal growth and production temperatures ranging from 18 to 30°C. The optimal germination temperatures for seed germination range from 25 to 30°C (Grubben & El Tahir, 2004).

According to Delelegn (2011), the production and consumption of peppers have progressively improved and increased globally in the 20th century owing to their roles as both spice and vegetables. Just like other plants in the family *Solanaceae* such as potatoes and tomato, peppers have increasingly become central components of various cuisines in the world as seen in the large acres of land dedicated to their cultivation in countries such as Mexico, China, India, USA, Korea, and Africa.

According to Bosland and Votava (2000), peppers, like their Solanaceous cousins, for example, eggplant and tomato, contain high amounts of vitamins C and A and are rich in vitamin B_2_, calcium, phosphorus, and potassium. Bosland and Votava (2000) reported increase in ascorbic acid and provitamin A content as hot peppers mature leading to extensive cultivation of pungent peppers for export market in several countries. A significant share of land in leading pepper‐producing nations is devoted for making chili powder. The increase in pepper industries at the international and national level as well as the ever‐rising demand of pepper to nourish the increasing human populace has necessitated the need for expanding cultivation of pepper in regions where cultivation has not been previously extensive (Beyene & David, 2007).

Pepper fruits contain various compounds such as capsaicinoids, carotenoids, fatty oils, steam volatile oil, protein, mineral elements, and vitamins among others (Amit Krishna De, 2003; Bosland & Votava, 2000). The mature fruits of pepper are particularly high in vitamin C, and the several other constituents of pepper have positive implication for nutritional value in human (Amit Krishna De, 2003; Marin, Ferreres, Tomas‐Barberan, & Gil, 2004). Capsaicinoids and carotenoids are the two main compounds in pepper of great importance and interests. Capsaicinoids are alkaloid compounds that give the characteristic pungency in hot chili peppers. They are bioactive molecules that are only produced in the genus *Capsicum* and are significant in food and medical sciences and in the defense weapon industry (Caterina et al., 2000; Chu et al., 2003). Although pepper seeds absorb capsaicin occasionally as a result of their proximity to the placenta, they are not the source of pungency Capsaicinoids are secreted only by the glands in fruit placenta. Carotenoids are pigments that confer the different colours found in the mature fruit of peppers and the rich supply of these pigments contribute to their nutritional value (Britton, Liaaen‐Jensen, & Pfander, 2009; Hornero‐Méndez, Costa‐García, & Mínguez‐Mosquera, 2002; Pérez‐López, del Amor, Serrano‐Martínez, Fortea, & Núñez‐Delicado, 2007).

## 5 *Capsicum annuum* (CHILI PEPPER)*:* ETHNOBOTANY, DISTRIBUTION, AND DESCRIPTION

*Capsicum annuum* L. includes a vast number of horticultural varieties (sweet and hot) and is by far the most important pepper economically. It is an important and popular vegetable in agriculture because of its economic importance and combination of taste, color, and nutritional values of the fruit (Al‐Snafi, 2015). *Capsicum annuum* comprises a range of bioactive compounds and essential nutrients that display various bioactivities such as antioxidant, antiviral, antimicrobial, anticancer, and anti‐inflammatory activities (Khan, Mahmood, Ali, Saeed, & Maalik, 2014). The place of origin of this species is Central and South America, and presently, it is extensively cultivated on above 1.5 million hectares in various countries especially throughout the tropical, subtropical, and temperate regions of Mexico, tropical Africa, East Africa, Europe, the Southern US, India, Turkey, China, and Ghana (FAO, 2007). *Capsicum annuum* grows in tropical climates, because of its requirement of a warm, humid climate in order to survive. It is a small herb which grows up to 1 m in height. The leaves are ovate, oblong‐ovate, or ovate‐lanceolate, 4–13 cm by 1.5–4 cm with entire margin, while the flowers are small, tinged purple, or white (Al‐Snafi, 2015). Most *Capsicum* fruits are red in color but several other types with green, orange, and yellow color exist. The seeds have pale yellow color, reniform, or discoid and are about 3–5 mm in size (Li, 2000). *Capsicum annuum* such as several plants require well‐drained, moisture‐holding loam soil that contains organic matter (Li, 2000). The optimal temperature requirement for growth and fruit production is in the range of 18–30°C, while the optimum temperature for seed germination is 25–30°C. Delayed flowering is encountered if daytime temperature is <25°C, and at night temperatures above 32°C, flower buds are aborted. Viability of the pollen is considerably reduced when temperatures are higher than 30°C and <15°C, while night temperatures down to 15°C favor setting of fruits. *Capsicum annuum* is day‐neutral; however, photoperiodic reaction may be shown in some forms. First flowering may be somewhat delayed by long days. Although flowering may be delayed by shade, shade up to 45% of solar radiation is tolerated. *Capsicum annuum* grows at several altitudinal ranges from lowland to 2,000 m, and it can be up to 3,000 m in Ethiopia. A minimum of 600 mm annual rainfall is needed when there is no irrigation. Water logging increases poor fruit setting, fruit rotting, and diseases. *Capsicum* is moderately sensitive to salinity of soil (Grubben & El Tahir, 2004).

## 6 POTENTIALS OF CHILI (*Capsicum annuum*) IN ALLEVIATING MICRONUTRIENT DEFICIENCIES

Studies by nutritionist have shown that the only sustainable approach that will enhance micronutrient status in humans is by the integration of foods high in micronutrients in the diet, and vegetables are an effective source of micronutrients based on the production cost and unit of land occupied. Vegetables also have advantages over legumes and cereals and in providing vitamins C and A as this is unavailable in these two sources (Ali & Tsou, 1997). The fruits of pepper are rich sources of capsaicinoids, carotenoids (with some of them having provitamin A activity), flavonoids, tocopherols (vitamin E), and ascorbic acid (vitamin C). The consumption of chili is rising, and this may represent an essential source of vitamins for the world populace. Several antioxidants, vitamins C, E, and provitamin A, are sufficiently available in high concentrations in several pepper types. Peppers apart from carotenoids are also good sources of xanthophylls and may contain high amounts of vitamins B, (riboflavin) B, (thiamine), B3 (niacin), and P (citrin), and are richer sources of vitamins A and C than the regularly recommended food sources (Bosland & Votava, 2000; Table 2).

**Table 2:** Vitamin content in *Capsicum annuum* (sweet/bell and green type)

Nutrient	Unit	1 value per 100 g
Vitamin C, total ascorbic acid	mg	80.6
Thiamin	mg	0.057
Riboflavin	mg	0.028
Niacin	mg	0.480
Pantothenic acid	mg	0.099
Vitamin B‐6	mg	0.224
Folate, total	μg	10
Folic acid	μg	0
Folate, food	μg	10
Folate, DFE	μg	10
Choline, total	mg	5.5
Betaine	mg	0.1
Vitamin B‐12	μg	0
Vitamin B‐12, added	μg	0
Vitamin A, RAE	μg	18
Retinol	μg	0
Carotene, beta	μg	208
Carotene, alpha	μg	21
Cryptoxanthin, beta	μg	7
Vitamin A, IU	IU	370
Lycopene	μg	0
Lutein + zeaxanthin	μg	341
Vitamin E (alpha‐tocopherol)	mg	0.37
Tocopherol, beta	mg	0
Tocopherol, gamma	mg	0
Tocopherol, delta	mg	0
Vitamin D (D2 + D3)	μg	0
Vitamin D	IU	0
Vitamin K (phylloquinone)	μg	7.4

### 6.1 The major vitamins in Chili

#### 6.1.1 Vitamin A

Globally, *Daucus carota* L. (Carrot) is thought to be the most important plant source of provitamin A carotenes. Although vitamin A is not found in peppers, high levels of the provitamins A including α‐carotene, β‐carotene, and β‐cryptoxanthin, which in the human liver can all be transformed into vitamin A, do occur. The most abundant form of provitamin A is α‐carotene, which cleaves to form two molecules of retinol, which is the physiologically active form of vitamin A (Yuni, Ana‐Rosa, Enny, Raoul, & Arnaud, 2013). In the world, protein and vitamin A deficiencies have been shown to be the dietary problems most occurring after total energy deficiency. Values of provitamin A are expressed as retinol activity equivalents (RAE), in which 1 RAE is equal to 12 μg of β‐carotene, 24 μg of α‐carotene, and 24 μg of β‐cryptoxanthin (Yuni et al., 2013). For men and women, recommended daily intake (RDI) values are 900 and 700 μg RAE/day, respectively. The levels of provitamin A per 100 g fresh weight of fruits are threefold to fivefold more in the red‐, orange‐, and brown‐fruited cultivars of chili than in yellow‐, purple‐, and green‐fruited cultivars and amount to 5%–10% RDI/100 g fresh weight edible portion (Yuni et al., 2013). The daily vitamin A requirement of an adult can be met by the consumption of just 3–4 g (about half a tablespoonful) of ground red pepper (Lantz, 1946). Also, several epidemiological studies have revealed that increased consumption of vitamin A or carotene can reduce cancer risk (Anon, 1982; Ziegler et al., 1986). Vitamin A (when it has been changed from provitamin A) is necessary for the differentiation of the conjunctival membranes, cornea, and retina, and for integrity of epithelial cells.

#### 6.1.2 Vitamin C

Vitamin C also referred to as ascorbic acid, a water‐soluble vitamin is an important antioxidant and a cofactor for enzymes that partake in metabolism of human (Yuni et al., 2013). The fruits of *Capsicum* as well as several other vegetables, such as cucumber, tomatoes, carrots, lettuce, and broccoli, provide high contents of vitamin C. Vitamin C contents in pepper genotypes vary between 43 and 247 mg/100 g of fresh fruits. This may increasingly give about 50% to over 100% RDI. The recent RDI for vitamin C in men and women is 90 and 75 mg/day, respectively (Yuni et al., 2013). A pepper fruit can contain six times as much vitamin C as an orange. Pepper fruits, from the green to the succulent red stage, each contain sufficient vitamin C that meet or exceed the adult RDA. Fresh fruits may contain up to 340 mg vitamin C mg/100 g. In pepper fruits, ascorbic acid level rises as the fruit ripens and this higher level noticed as it ripens could be attributed to intensity of light and high glucose levels which is the ascorbic acid precursor. Maturation as well as variations in environmental growing conditions and genetic background also affects the level of ascorbic acid in the fruit (Yuni et al., 2013).

#### 6.1.3 Vitamin E (Tocopherols)

According to Yuni et al. (2013), tocopherols are derivatives of (poly) isoprenoid, having a polar head group resulting from the metabolism of aromatic amino acid and a saturated tail formed from geranylgeranyl diphosphate. There are several forms of tocopherols which includes the α‐, β‐, γ‐, and δ‐forms, and their occurrence is based on the number and position of methyl groups on the aromatic ring. The different tocopherol compound contributes relatively to the total vitamin E activity. They also reported that α‐tocopherol has the utmost vitamin E activity, followed by β‐tocopherol, γ‐tocopherol, and δ‐tocopherol. Besides some other vegetables in the human diet such as broccoli, asparagus, eggplant, and cabbage, peppers are also in the list of the best sources of natural vitamin E. On a dry weight basis, dry red pepper powder has α‐tocopherol levels that are similar to those of spinach and asparagus and fourfold higher than that of tomatoes. The recommended daily intake of vitamin E is 15 mg/day of α‐tocopherol of both women and men. Pepper fruits can supply above 100% α‐tocopherol RDI per 100 g serving depending on the cultivar (Yuni et al., 2013).

### 6.2 Mineral contents in chili (*Capsicum annuum*)

Chilies are also excellent source of minerals such as molybdenum, manganese, foliate, potassium, and copper (Table 3). Ali and Tsou (1997), in their attempt to indicate that vegetables can supply more protein and iron in certain instances per unit of land per day more than cereals, standard micronutrients per 100 g of edible portion from various cereals and vegetables were multiplied with the various average per hectare per day yield of these crops grown under Taiwan conditions. In their report, pepper, when compared with cereals, produced double of the amount of iron from a given piece of land daily. Similarly, in their comparison of cereals and vegetables with regard to micronutrient supply per unit of production cost, pepper was reported to be 3.1 times more cost‐efficient in supplying iron than cereals. Ali and Tsou (1997), using an in vitro dialysis method, screened about 50 vegetables for their iron bioavailability and reported that the bioavailability of iron in 37 of 48 samples of vegetable was increased by cooking. They reported that a large range of differences in iron bioavailability between cooked and fresh samples existed, from 0.19% to 33.75%. In the cooked and fresh forms, chili peppers, tomatoes, ginger, and spinach were reported to have high iron content.

**Table 3:** Mineral content in *Capsicum annuum* (sweet/bell and green type)

Nutrient	Unit	1 value per 100 g
Calcium, Ca	mg	10
Iron, Fe	mg	0.34
Magnesium, Mg	mg	10
Phosphorus, P	mg	20
Potassium, K	mg	175
Sodium, Na	mg	3
Zinc, Zn	mg	0.13
Copper, Cu	mg	0.066
Manganese, Mn	mg	0.122
Selenium, Se	μg	0.0
Fluoride F	μg	2.0

### 6.3 Macronutrients in *Capsicum annuum*

In several studies, it has been revealed that *C. annuum* is a highly nutritive fruit that contains several macronutrients such as protein, carbohydrate, fats, and dietary fiber (Ismail, Anjum, Mamon, & Kazi, 2011; Tripathi & Mishra, 2009). The composition of macronutrients present in *C. annuum* is summarized in Table 4, and the nutrient present in the pungent type is summarized in Table 5.

**Table 4:** Proximate composition of *Capsicum annuum* (sweet/bell and green type)

Nutrient	Unit	1 value per 100 g
Water	g	93.89
Energy	kcal	20
Energy	kJ	84
Protein	g	0.86
Total lipid (fat)	g	0.17
Ash	g	0.43
Carbohydrate, by difference	g	4.64
Fiber, total dietary	g	1.7
Sugars, total	g	2.40
Sucrose	g	0.11
Glucose (dextrose)	g	1.16
Fructose	g	1.12
Lactose	g	0
Maltose	g	0
Galactose	g	0
Starch	g	0

**Table 5:** Nutritional composition of hot *Capsicum annuum* per 100 g

Nutrients	Amount
Macronutrients	
Water (g)	88
Calories (kcal)	40
Protein (g)	1.9
Carbohydrate (g)	8.8
Fiber (g)	1.5
Sugar (g)	5.3
Total Fat (g)	0.4
Micronutrients (Vitamins/Mineral)	
Vitamin A (μg)	45
Beta‐carotene (μg)	535
Vitamin B_6_ (mg)	0.51
Vitamin C (mg)	144
Iron (mg)	1
Magnesium (mg)	23
Potassium (mg)	322
Other constituent	
Capsaicin (g)	1–6

### 6.4 Pharmacological/Medicinal potentials of *Capsicum annuum*

Besides the nutritional benefits of pepper and their use as food additives, the hot *Capsicum* species (due to their capsaicin content) have a significant role in pharmacy and are currently used for different therapeutic purposes. The active principle in red peppers responsible for their medical and pharmacological use is the pungent alkaloid called capsaicin (Peña‐Alvarez, Ramírez‐Maya, & Alvarado‐Suárez, 2009). Capsaicin is the principal capsaicinoid in chili peppers that accounts for about 71% of the total capsaicinoids in majority of the pungent types, followed by dihydrocapsaicin (Caterina et al., 2000; Chu et al., 2003). According to Estrada, Bernal, Díaz, Pomar, and Merino (2002), the degree of pungency varies among different *Capsicum* spp. as capsaicin and dihydrocapsaicin contents could be influenced by numerous factors like the stage of development of the fruit and the environmental growth conditions. Several research works have revealed that the higher the amount of capsaicin, the hotter the pepper, and the more the antioxidant level (Caterina et al., 2000; Chu et al., 2003). Capsaicin is most esteemed because they give relief from sore throats and fever, relief from cold symptoms, and on digestive system, they have soothing effects. They enhance circulation, especially of cold hands and feet as a relief for hangover (Srinivasan, 2016). Also, they play a role as a heart stimulant in regulation of blood circulation and strengthening of the arteries, thereby possibly keeping down the risk of heart attacks. Capsaicin is the only known chemical constituent of *Capsicum* as a counter‐irritant for external use as an over‐the‐counter analgesic drug by the U.S. Food and Drug Administration (FDA). Various medical and pharmacological applications have been reported in chili because of their capsaicin contents. Some of them are described below.

### 6.5 Antioxidants properties

Antioxidants are micronutrients, which are known to protect the body cells and fight off free radicals in the body. They perform this function by disallowing oxidation process (Palevitch & Craker, 1995). Capsaicin can be effective as an antioxidant even when consumed for a short period. As reported by Lee, Kim, Yoon, and Lee (2003), the measured oxidative stress (as malondialdehyde) in the kidney, lung, liver, and muscle of Wistar rats treated with capsaicin (i.p., 3 mg/kg body weight) for 3 days consecutively decreased. Also, Kempaiah and Srinivasan (2004) reported the effect of capsaicin on the antioxidant level of erythrocytes and liver in rats having excess lipids. The reported reduction in oxidant stress was attributed to 0.015% capsaicin treatment in the diet which gave rise to hypotriglyceridemic effect which was expressed by reducing the high lipid peroxide content.

Additionally, pepper fruit is one of the richest sources of the three strongest antioxidant substances: vitamin C, carotenoids, and vitamin E, and an immense protection against oxidative agents would be enhanced by their intake (Palevitch & Craker, 1995).

### 6.6 Antidiabetic/anti‐inflammatory activity

Substance P, a neuropeptide released by capsaicin, has been shown to reverse diabetes in mice (Tsui, Razavi, Chan, Yantha, & Dosch, 2007). According to a report by Okumura, Tsukui, Hosokowa, and Miyashita (2012), the antidiabetic activities of capsaicin and caffeine on the blood glucose level of obese/diabetic model mice were found to be high in KK‐A(y) obese/diabetic mice. Recently, Srinivasan (2016) has reported that capsaicin as natural anti‐inflammatory compounds was effective as indomethacin (a nonsteroidal anti‐inflammatory drug) in suppressing aberrant crypt foci in rat.

### 6.7 Enhancement of micronutrient uptake

Capsaicin was studied for a potential impact on the absorption of calcium, iron, and zinc in the intestine by evaluating their uptake from intestines of rats pre‐fed for 8 weeks with spice compound. Sections of small intestine isolated from these rats were analyzed for ex vivo uptake of calcium, iron, and zinc, and higher intake of these micronutrients was observed in capsaicin‐fed rats (Prakash & Srinivasan, 2012a, 2012b). Capsaicin helped by increasing permeability and absorptive surface of the intestinal wall thereby enhancing the absorption of micronutrients.

### 6.8 Chemo‐preventive properties

Capsaicin has been studied widely for its cancer‐preventing properties. According to Arora, Gill, Chauhan, and Rana (2015), capsaicin repressed the growth of several malignant cell lines by induction of autophagy, cycle arrest, apoptosis, as well as by the suppression of metabolical activation at cellular level. Other clinical examination carried out in China and in Japan revealed that capsaicin directly suppresses the growth of leukemic cells (Ito et al., 2004). In a different experiment, capsaicin was also able to prompt apoptosis in human lung cancer cells (Lee, Krisanapun, & Baek, 2010).

## 7 INTEGRATION OF *Capsicum annuum* INTO THE DIET

### 7.1 Possibilities

The great potential in *C. annuum* in improving diet can be enhanced by raising consumer's awareness and also enhancing the genetics of pepper (Kantar et al., 2016). *Capsicum annuum* could contribute to a crucial portion of an integrated strategy, including supplementation of nutrient and fortification of food, for combating human dietary deficiency. Direct consumption of the available varieties and further intake of nutritionally improved varieties can be used in the global approach aimed at addressing human dietary deficiency (Kantar et al., 2016). Although lower amounts of pepper are consumed every day when compared to staples, such as wheat or maize, nutritionally enhanced peppers can contribute to a diverse and healthy diet as they are a perfectly fine way to get the 5–9 g servings of fruit and veggies that the USDA recommends that humans consume daily. A 100 g serving of raw pepper (roughly equal to half of a large bell pepper) exceeds the recommended daily value (RDV) 5,000 IU of vitamin A, the 60 mg RDV of vitamin C (FDA, 2016), and 50% of the 400 μg folic acid RDV set by the U.S Food and Drug Administration for a 2,000 calorie diet in adults and children over age 4 (FDA, 2016). *Capsicum annuum* fruits can be eaten dried, fresh, or processed form. Nonpungent *C. annuum* fruits, commonly referred to as sweet pepper, can be eaten raw in salads, but more usually it can be fried, cooked, or served together with other foods. The pungent types also called aromatic hot pepper should be eaten in very small quantities and are regarded as a spice for seasoning and stimulation of appetite. The hot peppers are also prepared into ketchup or spice mixtures for flavoring several food kinds (Grubben & El Tahir, 2004).

### 7.2 Challenges/concern

In its natural form, the hot *Capsicum annuum* variety can be harmful for the digestive system as it can cause burning sensation and internal stomach pain (Al‐Snafi, 2015). The hot varieties are generally not recommended for young children because they do not have well‐developed digestive system yet. As a general rule, hot chili should not be given to children under 2 years old. Once the child is big enough, it can be included in their diets gradually to obtain the nutritional benefits (Al‐Snafi, 2015).

Capsaicin in the pungent *Capsicum annuum* variety can also irritate or burn the eyes or hands; therefore, hands should be thoroughly washed after handling the peppers and caution to be taken about touching your hands to your eyes. Direct application on open wounds or abrasion or close to the eyes should be avoided. Pregnant women and lactating mothers or patients diagnosed with hypersensitivity should avoid consumption of hot *Capsicum annuum* (Al‐Snafi, 2015).

## 8 CURRENT STATUS OF CHILI PRODUCTION AND CONSUMPTION

**Figure 2:**
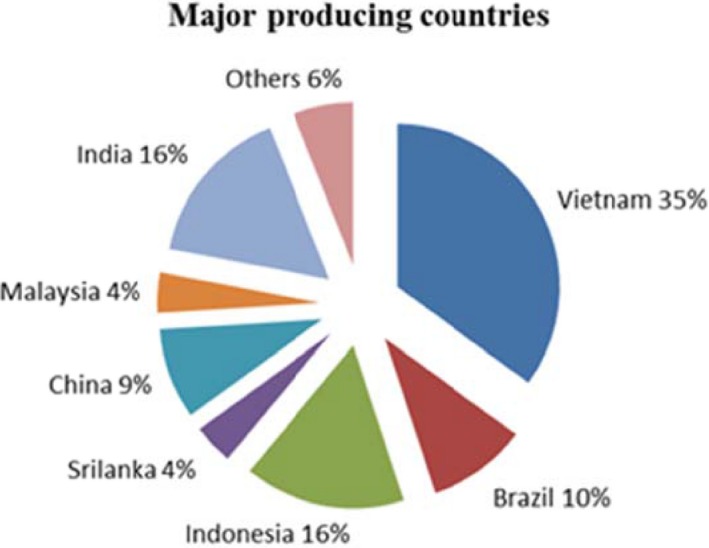
Percentage share of major *Capsicum annuum*‐producing countriesSource: International pepper community https://www.bing.com/images/search

**Figure 3:**
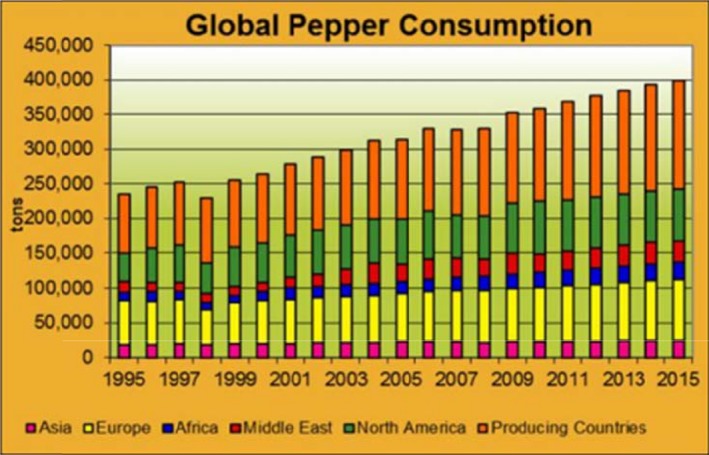
Global pepper consumptionSource: Nedspice (2015)

The 2014 global production of *C. annuum* in the world was approximated to be 409,000 tons, which is anticipated to rise to about 454,000 tons in 2015. Vietnam was on top of the list in 2014 with a yield of 155,000 tons which is about 35% share of global production (Nedspice, 2015). Major countries that grow chili are China, India, Pakistan, Korea, Indonesia, Turkey, and Sri Lanka in Asia; Ghana, Nigeria, Tunisia, and Egypt in Africa; Mexico and the United States in North and Central America; Spain, Yugoslavia, Bulgaria, Romania, Italy, and Hungary in Europe; and Peru, Argentina, and Brazil in South America. The global trade in chili accounts for 16% of the world's total spice trade (Nedspice, 2015) (Figures 2 and 3).

**Figure 4:**
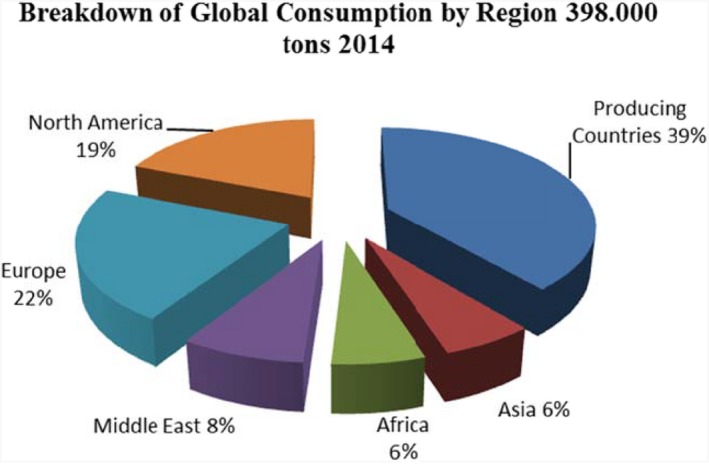
Breakdown of global consumption of pepper by regionSource: Nedspice (2015)

The global chili consumption is approximately 400,000 tons and has been rising progressively. Regional analysis is shown in Figure 4. China and India are the major consuming countries and are reported to consume about 49,000 and 59,000 mt, respectively, in 2014 (Nedspice, 2015). Consumption rate for Asia and the Middle East is 3%–4%. Globally, the rate of consumption is 2%–3% per annum. Despite the fact that China is a producer, it is noteworthy that their domestic consumption demand has risen above their production capacity in the last 10 years; therefore, they should be seen as a net consuming country. Sometimes, India has to import pepper to meet up with their local needs. Nevertheless, with conducive weather, India can meet local demand and still have excess to export (Nedspice, 2015).

## 9 PROBLEMS OF CHILI PRODUCTION AND POSSIBLE SOLUTION

Even though pepper is well planted all over the world, the obtained yields are usually very low (Adigun, 2001). Poor genetic potentials of most cultivars, use of poor seed quality, poor fruit quality due to lack of modern technical production skills, disease infestation, restriction of production to a single farming season, pervasive poverty, and low income among small holder farmers are some of the factors constraining pepper production by farmers which result to low yield (Fajinmi, 2006; Nedspice, 2015).

To get over this challenge, introduction of improved cultivars of pepper (with stimulating features such as disease resistance, high yielding potential, good quality, and wide acceptability of fruit color, taste, shape, and size) to farmers for evaluation and adoption is important. The high and regular market demand for pepper makes it important to enhance farmer's production capacity through introduction and promotion of improved varieties. Relatively, yield in developing countries is about 10%–30% of the yield in countries that are developed (Grubben & El Tahir, 2004).

Yield is variety‐dependent on varieties which in turn are well dependent on certain factors. For example, hot pepper production for dry fruit in Ethiopia has been low with a national average yield of 0.4 tons dry fruit yield/ha (Fekadu & Dandena, 2006). Lack of adaptable varieties with existing agro‐ecology and availability of water during dry seasons which can cause abortion of flower resulting in low productivity are responsible for the variation in yield. A lot of sustained efforts are being made to solve these production‐related constraints nationally and internationally.

Pepper yield enhancement programs have suggested that certain genotypes performed better than the others under some environmental conditions. Maintenance of soil richness has been proven to be a requirement for sustainable crop production and yield increase, while application of organic manure has been described to perform a vital role in this respect (Udoh, Ndon, Asuquo, & Ndaeyo, 2005). Application of organic manure has been said to improve productivity of the soil by increasing the micro‐organism in the soil, soil organic carbon content, soil structure improvement, the soil nutrient status, and enhancement of crop yield (Udoh et al., 2005).

In Africa, pepper yield improvement is achievable by the introduction and evaluation of more exotic varieties in multilocation trials (Udoh et al., 2005). Since yield depends on the genetic background of the plant and the environment, attention should be on the varieties that can at least moderately withstand prevailing environmental conditions like high temperatures, pathogens such as viruses, and bacteria associated with bacterial wilt. Promotion and adoption of best cultural practices are also necessary to lessen gap in pepper yield. In order to develop a better cropping system to achieve a high and stable productivity, the knowledge of the relationship between yield and environmental climatic factors is needed (Udoh et al., 2005).

## 10 CONCLUSION

A number of approaches are being employed to continue the fight against human dietary deficiency in developing countries where poor households are the most vulnerable. In many of these countries especially in the tropics, the rural people traditionally cultivate and harvest extensive varieties of vegetables. Food quality depends on the presence of various micro‐ and macronutrients. From the present review, it can be concluded that peppers are rich and economical vegetable that contains an appreciable amount of essential micro‐ and macronutrients that are nutritionally valuable and also contain healthy ingredients to promote health. The presence of phytochemicals and antioxidants in pepper is also important in the prevention of chronic diseases. Therefore, integrating a pepper‐rich diet in our daily meals can be helpful in the continuing quest to alleviate micronutrient deficiency.

## CONFLICT OF INTEREST

The authors declare that they do not have any conflict of interest.

## ETHICAL REVIEW

This review does not involve any human or animal testing.
